# Comparison of Anorectic Potencies of Type A Trichothecenes T-2 Toxin, HT-2 Toxin, Diacetoxyscirpenol, and Neosolaniol

**DOI:** 10.3390/toxins10050179

**Published:** 2018-04-29

**Authors:** Jie Zhang, Hua Zhang, Shengli Liu, Wenda Wu, Haibin Zhang

**Affiliations:** 1 College of Veterinary Medicine, Nanjing Agricultural University, Nanjing 210095, China; tingyuxuansanhao@163.com (J.Z.); 2016207033@njau.edu.cn (H.Z.); 2 Shandong Lonct Enzymes Co., Ltd., Linyi 276000, China; LSL200660@126.com

**Keywords:** trichothecene, anorexia, T-2 toxin, HT-2 toxin, diacetoxyscirpenol, neosolaniol

## Abstract

Trichothecene mycotoxins are common contaminants in cereal grains and negatively impact human and animal health. Although anorexia is a common hallmark of type B trichothecenes-induced toxicity, less is known about the anorectic potencies of type A trichothecenes. The purpose of this study was to compare the anorectic potencies of four type A trichothecenes (T-2 toxin (T-2), HT-2 toxin (HT-2), diacetoxyscirpenol (DAS), and neosolaniol (NEO)) in mice. Following oral exposure to T-2, HT-2, DAS, and NEO, the no observed adverse effect levels (NOAELs) and lowest observed adverse effect levels (LOAELs) were 0.01, 0.01, 0.1, and 0.01 mg/kg body weight (BW), and 0.1, 0.1, 0.5, and 0.1 mg/kg BW, respectively. Following intraperitoneal (IP) exposure to T-2, HT-2, DAS, and NEO, the NOAELs were 0.01 mg/kg BW, except for DAS (less than 0.01 mg/kg BW), and the LOAELs were 0.1, 0.1, 0.01, and 0.1 mg/kg BW, respectively. Taken together, the results suggest that (1) type A trichothecenes could dose-dependently elicit anorectic responses following both oral gavage and IP exposure in mice; (2) the anorectic responses follow an approximate rank order of T-2 = HT-2 = NEO > DAS for oral exposure, and DAS > T-2 = HT-2 = NEO for IP administration; (3) IP exposure to T-2, HT-2, DAS, and NEO evoked stronger anorectic effects than oral exposure. From a public health perspective, comparative anorectic potency data should be useful for establishing toxic equivalency factors for type A trichothecenes.

## 1. Introduction

**Figure 1:**
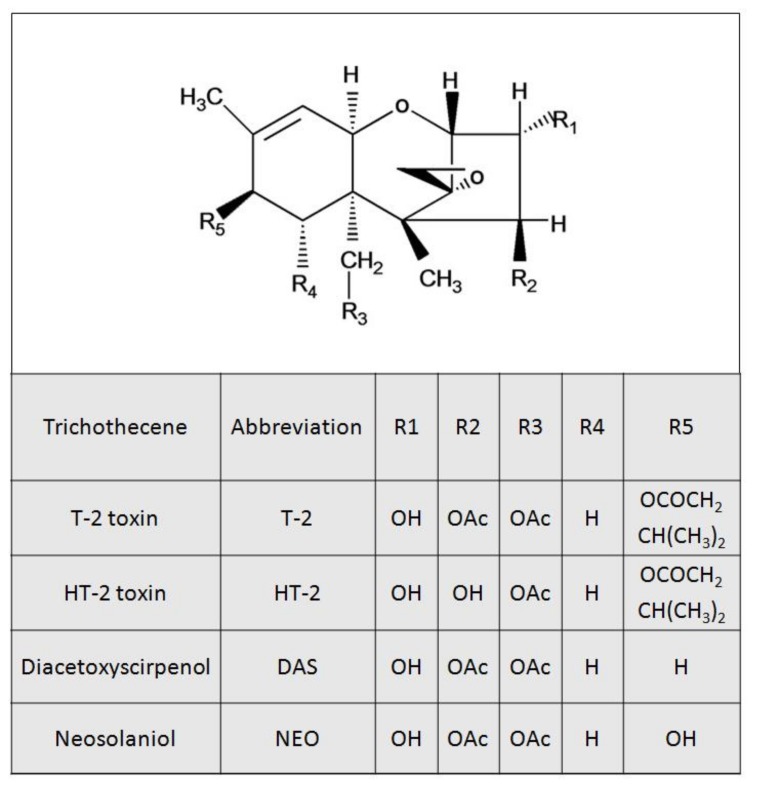
Structures of Type A trichothecenes.

Trichothecene mycotoxins are secondary metabolites of *Fusarium* and are widely distributed around the world [1]. Generally, these toxins have a common tetracyclic 12, 13-epoxytrichothecene skeleton, and can be classified into A, B, C, and D categories [2]. Due to their strong toxicity and high contamination rate, type A and B trichothecenes are of particular public health concern, respectively [3,4]. The type A trichothecenes mainly include T-2 toxin (T-2), HT-2 toxin (HT-2), diacetoxyscirpenol (DAS), and neosolaniol (NEO) (Figure 1). A spectrum of adverse effects including anorexia, emesis, growth suppression, neurotoxicity, immunotoxicity, genotoxicity, and hepatotoxicity were observed following exposure to type A trichothecenes [5,6,7,8,9,10]. T-2 is considered to be the most toxic of the trichothecenes [11,12]. T-2s capacity to cause animal toxicosis is mentioned in many countries, including USA, Canada, and Japan [13,14,15]. A T-2 food poisoning outbreak in humans has been reported in China [16]. T-2 is mainly metabolized into HT-2, which has comparable toxicity with T-2 [17,18]. A small proportion of T-2 is also metabolized into DAS and/or NEO, and these two toxins are less toxic than T-2 [19,20].

The potential for anorexia induction by type A trichothecenes through targeting the appetite center is of particular concern from the perspective of human and animal health [21]. T-2-induced anorexia has been observed in many animal species following oral, intraperitoneal (IP), intubation, intravenous (iv), intracerebroventricular (icv), sublingual, and subcutaneous (sc) exposure routes. Both oral and sc administrations of T-2 induce anorexia and vomiting in cats [22]. Severe food refusal is observed in sheep after consuming T-2-contaminated cereal grain [23]. Following intubation with 0.6 mg/kg BW T-2 in Holstein cows, marked anorectic responses were observed [24]. T-2 induces significant food refusal in rodents following both oral gavage and icv injection [25,26]. Due to the comparable toxicity, HT-2 elicits analogous capacity to T-2 in inducing anorexia [19]. Anorectic responses are reported in pigs and broiler chicks following intubation with DAS at 2 and 3.82 mg/kg BW, respectively [27,28]. However, there is no relevant research on regulation of food consumption by NEO.

To date, the anorectic potencies of the type A trichothecenes T-2, HT-2, DAS, and NEO are not well characterized within one species. To address this gap, we compared the anorectic potencies of T-2, HT-2, DAS, and NEO using a mouse bioassay developed previously [29]. The results indicated that (1) type A trichothecenes could dose-dependently elicit anorectic responses following both oral gavage and IP exposure in mice; (2) the anorectic responses follow an approximate rank order of T-2 = HT-2 = NEO > DAS for oral exposure, and DAS > T-2 = HT-2 = NEO for IP administration; (3) IP exposure to T-2, HT-2, DAS, and NEO evoked stronger anorectic effects than oral administration.

## 2. Results

### 2.1. Dose Dependent Effects of T-2 Toxin, HT-2 Toxin, DAS and NEO on Food Intake Following Oral Gavage

**Figure 2:**
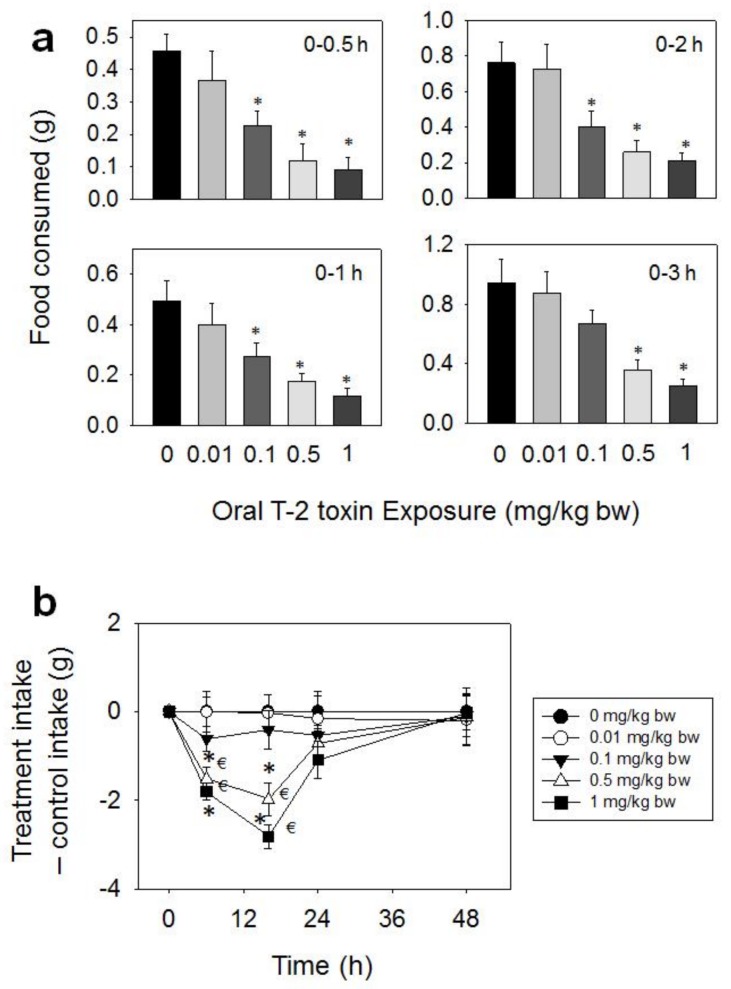
Oral exposure to T-2 toxin (T-2) impairs cumulative food intake for up to 48 h. (**a**) Short term (0–3 h) food refusal induced by oral T-2 toxin exposure. Data are mean ± SEM (*n* = 6/gp). Food intake at specific time points was analyzed by one-way ANOVA using Dunnett’s Test to determine significant differences between an individual treatment and the vehicle control. Symbol: * indicates difference in food consumption as compared to the control (*p* < 0.05) for a given time period; (**b**) Long term (0–96 h) food refusal induced by oral T-2 toxin exposure. Data are mean ± SEM (*n* = 6/gp). Two-way repeated ANOVA (one-factor) using Bonferroni t-test was used to analyze significant differences in food consumption as compared to the control over time. Symbols: * indicates difference in cumulative food consumption relative to the control at specific time point (*p* < 0.05), and € indicates difference in cumulative food consumption relative to the 0.5 h time point within a given dose (*p* < 0.05).

Following oral administration with T-2 toxin, significant reductions in food intake were observed at doses 0.1, 0.5, and 1 mg/kg BW during 0.5–48 h, whereas effects were not seen for 0.01 mg/kg BW (Figure 2a,b). The 0.1 mg/kg BW dose induced 51, 46, and 47% reductions in cumulative food intake at 0.5, 1, and 2 h, respectively, with no differences being observed after 3 h. Reduced food intake was observed at 0.5 mg/kg BW after 0.5 (74%), 1 (64%), 2 (66%), 3 (62%), 6 (62%), and 16 h (46%), respectively, with no differences being observed after 24 h. The 1 mg/kg BW dose evoked 80, 76, 72, 73, 78, and 68% reductions in cumulative food intake at 0.5, 1, 2, 3, 6, and 16 h , respectively. From 16 h to 48 h, there was a trend toward increased food consumption, thus compensating for initial food refusal by 48 h.

**Figure 3:**
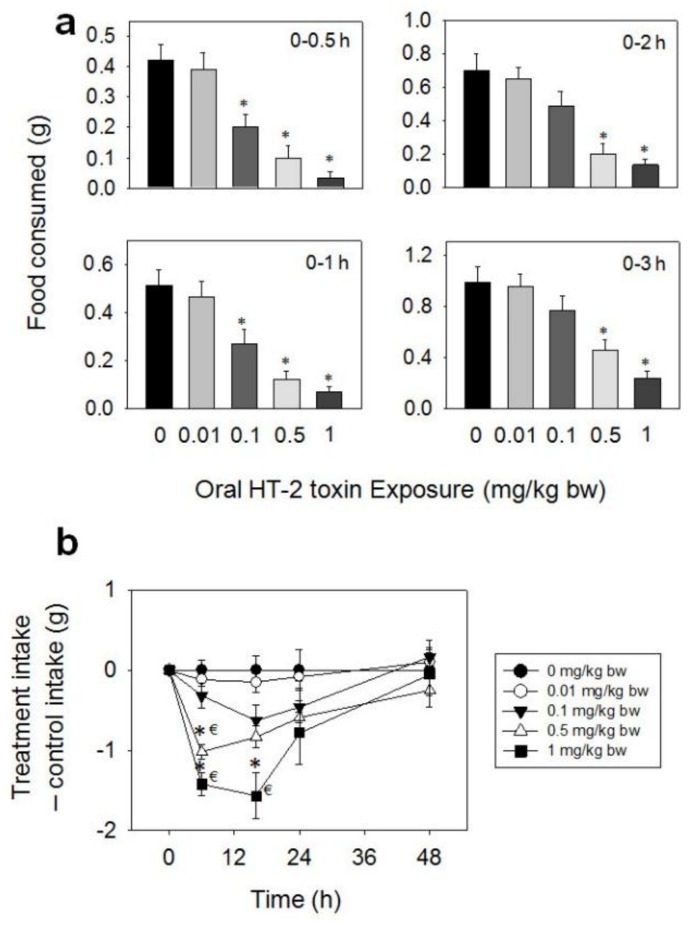
Oral exposure to HT-2 toxin (HT-2) impairs cumulative food intake for up to 48 h. (**a**) Short term (0–3 h) food refusal induced by oral HT-2 toxin exposure; (**b**) Long term (0–96 h) food refusal induced by oral HT-2 toxin exposure. Data are mean ± SEM (*n* = 6/gp). The experiment was conducted and data analyzed as described in the Figure 2 legend.

When the anorectic effects of oral exposure to HT-2 toxin were evaluated, marked reductions in food intake were observed at 0.1, 0.5, and 1 mg/kg BW during 0.5–48 h, while no effect was observed at 0.01 mg/kg BW dose (Figure 3a,b). At the 0.1 mg/kg BW dose, 52% and 47% reductions in cumulative food consumption were observed at 0.5 and 1 h, respectively, with no differences being observed after 2 h. Marked reductions in cumulative food intake were observed after 0.5 (77%), 1 (76%), 2 (71%), 3 (54%), and 6 h (53%) at 0.5 mg/kg BW, respectively, with no differences being observed after 16 h. The 1 mg/kg BW dose caused 92, 87, 81, 76, 73, and 60% reductions in cumulative food intake at 0.5, 1, 2, 3, 6, and 16 h, respectively. There was a trend toward increased food consumption from 16 h to 48 h, which compensated for initial food refusal by 48 h.

**Figure 4:**
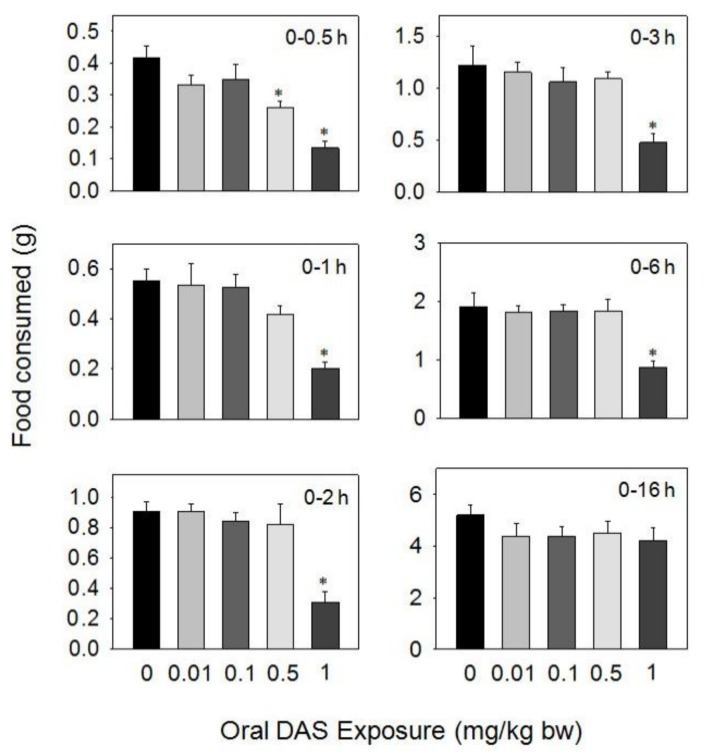
Oral exposure to diacetoxyscirpenol (DAS) impairs cumulative food intake for up to 16 h. Data are mean ± SEM (*n* = 6/gp). The experiment was conducted and data analyzed as described in the Figure 2 legend.

Upon oral dosing with DAS at 0.5 and 1 mg/kg BW during 0.5–16 h, food intake was significantly reduced, respectively, compared to the control, but was not affected by doses of 0.01 and 0.1 mg/kg BW of the toxin (Figure 4). A marked reduction in food intake was observed only after 0.5 h (38%) at 0.5 mg/kg BW, with no differences being observed after 1 h. The 1 mg/kg BW dose inhibited food intake by 68%, 63%, 65%, 59%, and 54% at 0.5, 1, 2, 3, and 6 h, respectively, but no inhibition was observed at 16 h post-treatment.

**Figure 5:**
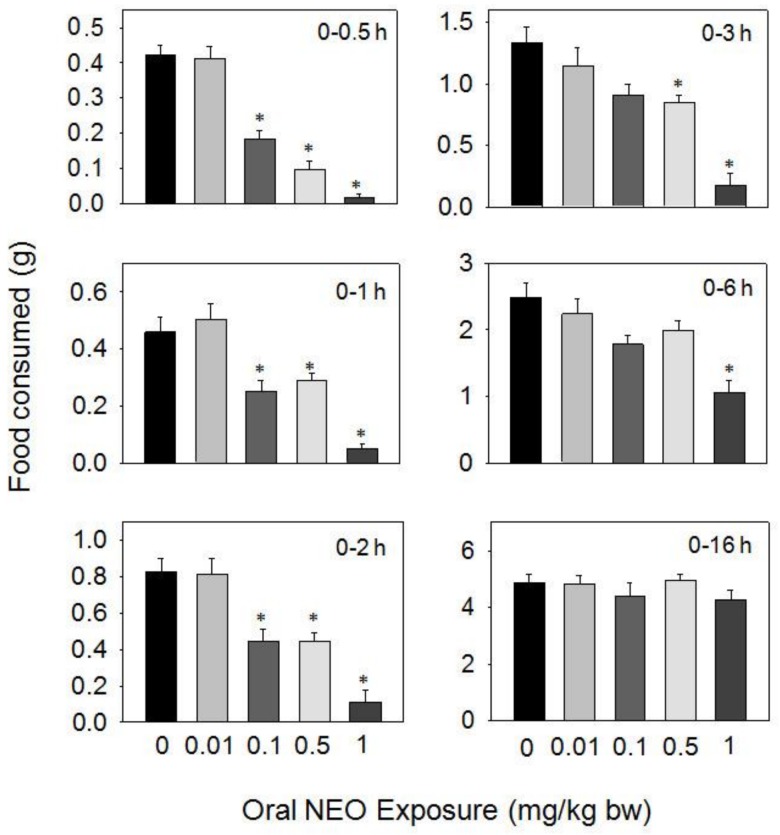
Oral exposure to neosolaniol (NEO) impairs cumulative food intake for up to 16 h. Data are mean ± SEM (*n* = 6/gp). The experiment was conducted and data analyzed as described in the Figure 2 legend.

Oral exposure to NEO caused reductions in food consumption at 0.1, 0.5, and 1 mg/kg BW during 0.5–16 h, but 0.01 mg/kg BW had no effect (Figure 5). The 0.1 mg/kg BW dose resulted in reduced food consumption of 56, 46, and 45% at 0.5, 1, and 2 h, respectively, with no differences being observed after 3 h. The 0.5 mg/kg BW dose caused 77, 38, 46, and 37% reductions in food consumption at 0.5, 1, 2, and 3 h, respectively, with no differences being seen after 6 h. For 1 mg/kg BW, dramatic reductions in food consumption were observed after 0.5 (96%), 1 (90%), 2 (87%), 3 (86%), and 6 h (58%), respectively, but no inhibition was observed at 16 h post-dosing.

### 2.2. Dose-Dependent Effects of T-2 Toxin, HT-2 Toxin, DAS, and NEO on Food Intake with IP Method

**Figure 6:**
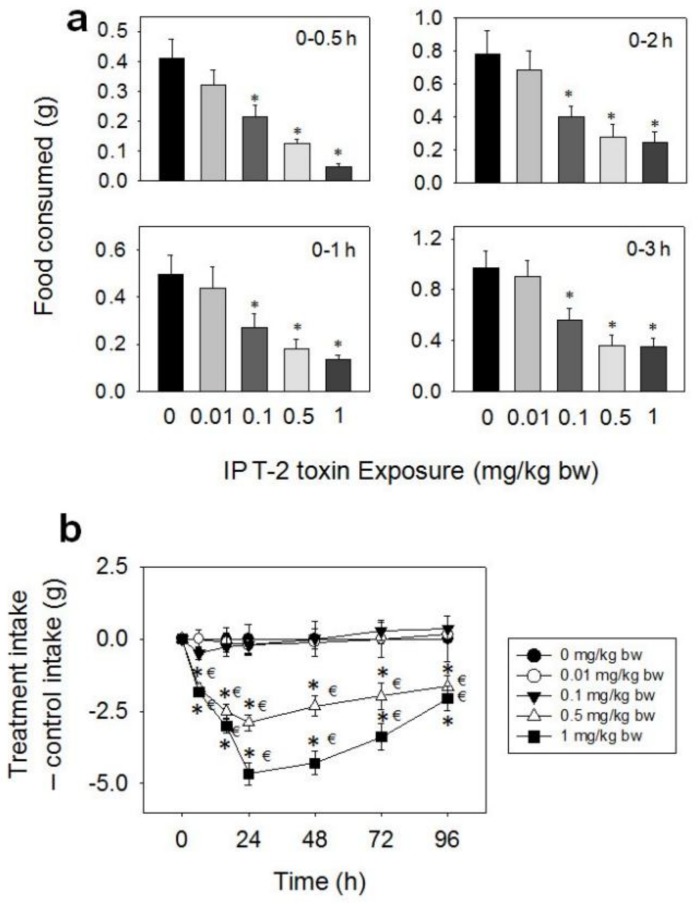
Intraperitoneal (IP) exposure to T-2 toxin impairs cumulative food intake for up to 96 h. (**a**) Short term (0–3 h) food refusal induced by IP T-2 toxin exposure; (**b**) Long term (0–96 h) food refusal induced by IP T-2 toxin exposure. Data are mean ± SEM (*n* = 6/gp). The experiment was conducted and data analyzed as described in the Figure 2 legend.

After IP exposure to T-2 toxin at 0.1, 0.5, and 1 mg/kg BW, decreased food consumption was observed from 0.5–96 h, while no effect was observed at the 0.01 mg/kg BW dose (Figure 6a,b). At the 0.1 mg/kg BW dose, cumulative food intake reductions of 48, 45, 46, and 41% were observed at 0.5, 1, 2, and 3 h, respectively, with no differences being observed after 6 h. Dramatic reductions in food intake were observed after 0.5 (70 and 88%), 1 (63 and 73%), 2 (65 and 68%), 3 (63 and 64%), 6 (77 and 80%), 16 (63 and 74%), and 24 h (57 and 70%) at 0.5 and 1 mg/kg BW, respectively. From 24 h to 48 h, there was a trend toward increased food consumption at 0.5 and 1 mg/kg BW doses. However, these two groups did not fully compensate for initial food refusal by 96 h.

**Figure 7:**
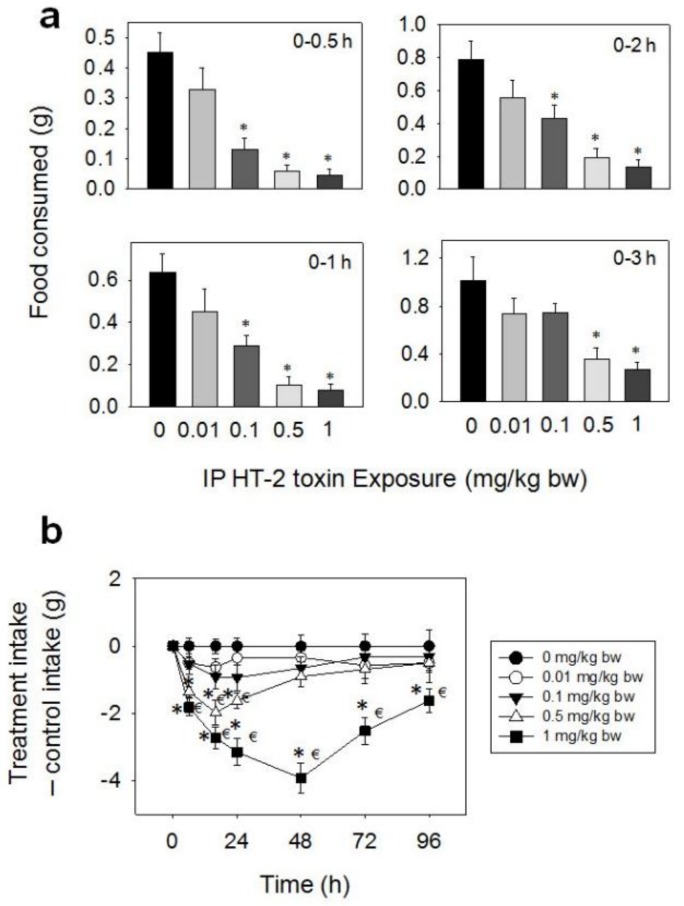
IP exposure to HT-2 toxin impairs cumulative food intake for up to 96 h. (**a**) Short term (0–3 h) food refusal induced by IP HT-2 toxin exposure; (**b**) Long term (0–96 h) food refusal induced by IP HT-2 toxin exposure. Data are mean ± SEM (*n* = 6/gp). The experiment was conducted and data analyzed as described in the Figure 2 legend.

Food intake was reduced in mice after IP exposure to HT-2 toxin at 0.1, 0.5, and 1 mg/kg BW during 0.5–96 h, but 0.01 mg/kg BW had no effect (Figure 7a,b). Significant reductions in cumulative food consumption were observed only at 0.5 (71%), 1 (55%), and 2 h (45%) after exposure to 0.1 mg/kg BW, with no differences being observed between the control and 0.1 mg/kg BW groups after 3 h. The 0.5 mg/kg BW dose caused 87, 84, 75, 64, 63, 51, and 36% reduced cumulative food intake at 0.5, 1, 2, 3, 6, 16, and 24 h, respectively, with no differences being observed after 48 h. The 1 mg/kg BW dose evoked 90, 87, 83, 73, 78, 69, 65, and 40% reductions in cumulative food intake at 0.5, 1, 2, 3, 6, 16, 24, and 48 h, respectively. 0.5, 1, 2, 3, 6, 16, 24, and 48 h, respectively. From 48 h to 96 h, there was a trend toward increased food consumption. However, by 96 h, this group still did not fully compensate for initial food refusal.

**Figure 8:**
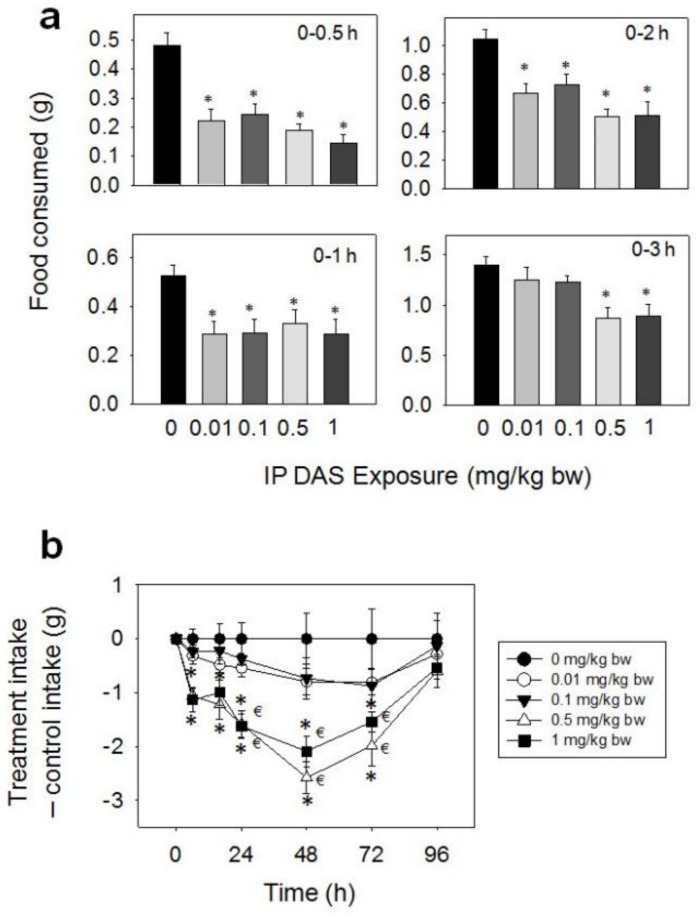
IP exposure to DAS impairs cumulative food intake for up to 96 h. (**a**) Short term (0–3 h) food refusal induced by IP DAS exposure; (**b**) Long term (0–96 h) food refusal induced by IP DAS exposure. Data are mean ± SEM (*n* = 6/gp). The experiment was conducted and data analyzed as described in the Figure 2 legend.

After IP exposure of mice to 0.01, 0.1, 0.5, and 1 mg/kg BW DAS, marked reductions in food intake were observed at 0.5 (54, 50, 61 and 70%), 1 (46, 45, 39 and 47%), and 2 h (36, 30, 52 and 51%), respectively (Figure 8a,b). At 3 h post-exposure and thereafter, differences were no longer observed between control and 0.01 or 0.1 mg/kg BW. For the 0.5 and 1 mg/kg BW doses, cumulative food intake was reduced after 3 (38 and 37%), 6 (49 and 51%), 16 (48 and 45%), 24 (43 and 43%), and 48 h (40 and 35%), as compared with control mice. From 48 h to 96 h, there were trends toward increased food consumption at the 0.5 and 1 mg/kg BW doses, thus compensating for initial food refusal by 96 h.

**Figure 9:**
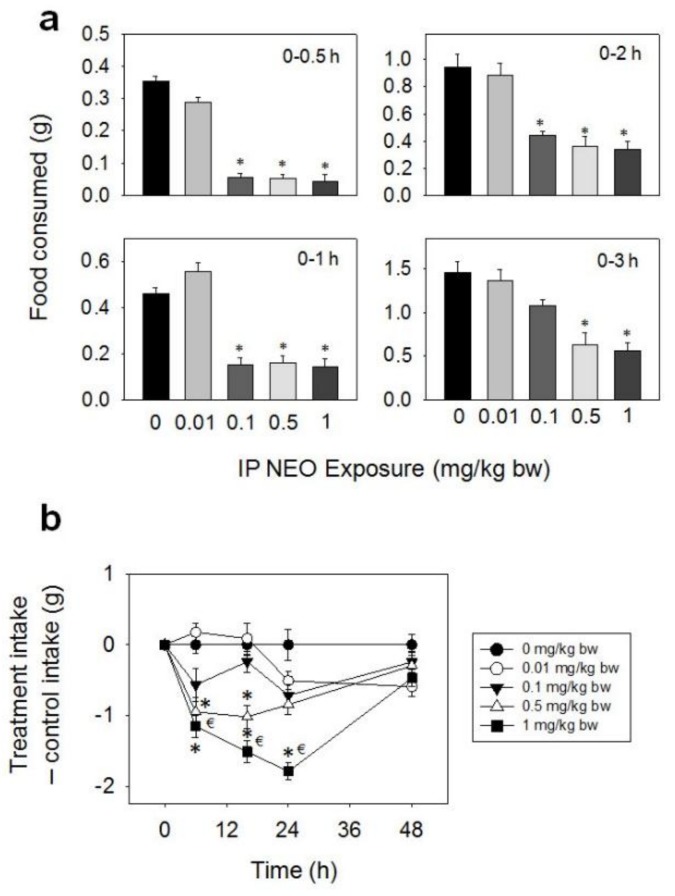
IP exposure to NEO impairs cumulative food intake for up to 48 h. (**a**) Short term (0–3 h) food refusal induced by IP NEO exposure; (**b**) Long term (0–96 h) food refusal induced by IP NEO exposure. Data are mean ± SEM (*n* = 6/gp). The experiment was conducted and data analyzed as described in the Figure 2 legend.

Following IP administration with NEO, significant reductions in food intake were observed at 0.1, 0.5, and 1 mg/kg BW from 0.5–48 h, whereas effects were not seen for 0.01 mg/kg BW (Figure 9a,b). The 0.1 mg/kg BW dose caused 84, 67, and 54% reductions in food intake at 0.5, 1, and 2 h, respectively, with no differences being seen after 3 h. For the 0.5 mg/kg BW dose, marked reductions in food consumption were observed after 0.5 (85%), 1 (66%), 2 (62%), 3 (57%), 6 (41%), and 16 h (32%), with no differences being observed after 24 h. The 1 mg/kg BW dose evoked 88, 69, 64, 62, 50, 43, and 30% reductions in cumulative food intake at 0.5, 1, 2, 3, 6, 16, and 24 h, respectively. There was a trend toward increased food consumption from 24 h to 48 h, and by 48 h this compensated for initial food refusal.

## 3. Discussion

Although type A trichothecene-induced anorexia constitutes major concern for human and animal health, there have been relatively few studies of their comparative effects in a common animal model. This study is novel because it is the first investigation to compare the anorectic potencies of four major type A trichothecenes, T-2, HT-2, DAS, and NEO, in an established mouse anorexia bioassay. Herein, we revealed that T-2, HT-2, DAS, and NEO elicited anorectic effects with different durations in a dose-dependent fashion. IP exposure to these toxins caused more severe anorectic responses than oral gavage, based on dose and duration.

The no observed adverse effect levels (NOAELs) and lowest observed adverse effect levels (LOAELs) for T-2, HT-2, DAS, and NEO following oral and IP administration were summarized in Table 1. These data indicated that the anorectic potencies of type A trichothecenes generally follow rank orders of T-2 = HT-2 = NEO > DAS for oral exposure, and DAS > T-2 = HT-2 = NEO for IP administration. It was further notable that the NOAELs and LOAELs of T-2 and HT-2 in the present study (0.01 and 0.1 mg/kg BW for both oral and IP exposure) were identical with our previous investigation [19], but differ somewhat from those reported previously in studies with other species. For example, it has reported that the NOAEL and LOAEL for T-2-induced anorectic responses following oral exposure in rats were 1 and 3 mg/kg BW, respectively [30]. Another study reported that the anorectic LOAEL for oral exposure to T-2 in rabbits was 2 mg/kg BW [31]. In cats, the anorectic LOAELs of T-2 were 0.08 and 0.05 mg/kg BW following oral and sc exposure, respectively [22,32]. Differences in animal species and experimental design in absorption, distribution, metabolism, and excretion of T-2 could explain the differences between these prior studies and the current investigation. In this study, the dose-dependency of T-2 and HT-2-induced anorexia was analogous. Because T-2 is almost completely metabolized to HT-2 after exposure, the possible reason for the equivalent anorectic potencies of the two toxins might be efficient biotransformation of T-2. [17,19,33,34,35]. The anorectic NOAELs and LOAELs of DAS and NEO following oral and IP exposure presented here were the first published values for any species.

In this study, anorectic responses induced by T-2 and HT-2 could rapidly occur within 0.5 h, and were sustained up to 96 h. Intubating with T-2 (3-^3^H-labeled) in broiler chicks, the GI tract contained 88.8% of the recovered radioactivity within 0.5 h [36]. Pace and co-workers indicated that 75% of the T-2 (3-^3^H-labeled) was excreted in feces and urine within five days, and the toxic responses induced by T-2 dribble away over time [37]. Rapid absorption and longer elimination may lead to the onset and duration of anorectic effects induced by these toxins. The 0.5 mg/kg BW dose of DAS induced anorexia that was only observed within 0.5 h following oral gavage, while the 0.01 mg/kg BW dose could cause an anorectic response within 0.5 h, and sustain for 2 h with IP administration. It was notable that the dosage of 0.01 mg/kg BW was lower than other type A trichothecenes. The results indicated that the capacity of DAS in inducing anorexia was relatively weaker than that of T-2, HT-2, and NEO with the oral method, but higher than that of T-2, HT-2, and NEO with IP exposure [27,28]. However, the related mechanism of these results still need further research. The DAS-induced anorectic response rapidly disappeared after 6 h, due to approximately 90% of the DAS being excreted in urine and feces during the first 24 h following the oral administration method [38]. The anorectic responses induced by NEO were observed within 0.5 h, and were sustained for 6 h and 24 h following oral gavage and IP administration, respectively. Although the durations of anorexia induced by NEO were shorter than those of T-2 and HT-2, the NOAELs and LOAELs of these three toxins were identical. Hence, we indicate that T-2, HT-2, and NEO possess similar capacities in causing anorectic responses following oral gavage and IP injection in mice.

Appetite regulation is a complex process and involves many factors, including gut satiety hormones, neurotransmitters, and pro-inflammatory cytokines. Gut satiety hormones are well-characterized in anorexia induction by trichothecenes. DON could elevate the levels of gut satiety hormones in the plasma, including cholecystokinin (CCK), peptide YY_3-36_ (PYY_3-36_), glucagon-like peptide-1 (GLP-1), and glucose dependent insulinotropic polypeptide (GIP) in mice [39,40,41,42]. Inhibiting the receptors of these hormones could attenuate DON-induced anorexia, suggesting that these hormones are involved in DON-induced anorexia. Similarly, elevations of plasma CCK, PYY_3-36_, GLP-1, and GIP, as well as correlated anorectic responses, were observed in mice following oral and IP administrated with T-2, HT-2, DAS, and NEO [43,44]. Blocking hormone receptors also attenuated anorexia induced by T-2, indicating that these hormones also play important roles in T-2-induced anorexia [45]. In addition to satiety hormones, neurotransmitter 5-hydroxytryptamine (5-HT) is also involved in appetite regulation [46,47]. Following IP and oral administration of DON and T-2, a dramatic increase of 5-HT was observed in minks [48,49]. Food refusal and intestinal motility inhibition induced by DON are mediated through the 5-HT3 receptor [50]. Similarly, 5-HT has been shown to contribute to the T-2-, HT-2-, DAS-, and NEO-induced anorectic responses (unpublished).

Trichothecenes are well known to upregulate the expression of pro-inflammatory cytokines, including interleukin-1β (IL-1β), interleukin-6 (IL-6), and tumor necrosis factor-α (TNF-α) [39]. Such a cytokine storm could cause sickness, with anorexia as a hallmark [51]. Elevations of IL-1β, IL-6, and TNF-α were observed in DON-administered mice that were correlated to DON-induced anorexia [39,52]. Both IL-1β and TNF-α receptor antagonists have the potency to attenuate DON-induced anorectic responses [53]. DON can markedly upregulate the hypothalamic mRNA levels of the anorexigenic modulator pro-opiomelanocortin (POMC), as well as IL-1β, IL-6, and TNF-α [54]. Gaigé and co-workers indicated that T-2-induced anorexia correlated with IL-1β, IL-6, and TNF-α mRNA upregulation in the spleen and liver [25]. Therefore, it might be speculated that anorexia induction by trichothecenes could be mediated by the pro-inflammatory cytokines IL-1β, IL-6, and TNF-α as part of sickness behavior.

It was particularly notable that the brain was the target organ of trichothecenes [55,56]. Some investigations have demonstrated that both DON and T-2 could activate c-Fos expression in circumventricular organs and surrounding structures of the brain [25,54]. Usually, c-Fos expression in the brain results from activating the brainstem/hypothalamus connecting networks, or the vagus nerve. It is possible that toxins could directly regulate anorectic neurocircuitry in the brain and lead to anorexia. DON was observed to rapidly enter the brain within 5 min after exposure [57], supporting this speculation. Another possibility is that toxins activate anorectic neurocircuitry in the brain indirectly through the vagus nerve. Girardet and co-workers indicated that vagotomy had no impact on DON-induced c-Fos expression, suggesting DON has the potency to enter and impact the brain directly [54]. In contrast, T-2-induced c-Fos expression was attenuated by vagotomy [25]. This suggests that T-2 is capable of acting indirectly in the brain through the vagus nerve.

## 4. Conclusions

To summarize, the results presented herein indicated that (1) the type A trichothecenes T-2, HT-2, NEO, and DAS could dose-dependently elicit anorectic responses following both oral gavage and IP exposure in mice; (2) the anorectic responses follow an approximate rank order of T-2 = HT-2 = NEO > DAS for oral exposure, and DAS > T-2 = HT-2 = NEO for IP administration; (3) IP exposure to T-2, HT-2, DAS, and NEO evoked stronger anorectic effects than oral exposure. From the health perspectives of both human and animals, this study will improve our understanding of the risks of type A trichothecenes and facilitate the formulation of strategies about prophylaxis.

## 5. Materials and Methods

### 5.1. Chemicals

T-2, HT-2, DAS, and NEO were obtained from Sigma-Aldrich (St.Louis, Missouri, USA) (purity ≥ 98%, High Performance Liquid Chromatography) and dissolved in 1% dimethylsulfoxide (DMSO) in filter-sterilized phosphate buffered saline (PBS). Each toxin was diluted with PBS for 0.01, 0.1, 0.5, and 1 mg/kg BW.

### 5.2. Animals

Female B6C3F1 mice (average weight = 20 ± 2 g) were obtained from Vital River Laboratories (Beijing, China). A total of 252 mice were housed individually in polycarbonate cages in a room maintained at 21–24 °C and 40–55% relative humidity under a 12 h light (6:00–18:00 h)/dark (18:00–6:00 h) cycle. A high fat diet (45 kcal% fat diets, Jiangsu Medicine Company, Yangzhou, China), placed in 2-inch-high glass jars, was employed for the feeding bioassay, and sifted aspen chips were used for bedding. All experiments and protocols used in this study were approved by the Nanjing Agricultural University Institutional Animal Care and Use Committee (Certification No.: SYXK (Su) 2017-0007). Approval date: 2017.02.15 – 2022.02.14.

### 5.3. Experimental Design

**Figure 10:**
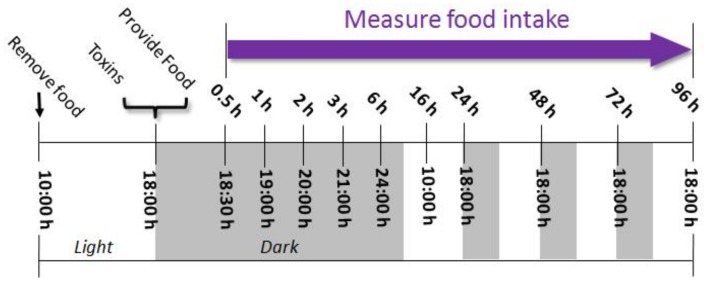
Experimental design for anorexia bioassay in mice. Mice were administered Type A trichothecenes by oral gavage or IP injection immediately before the dark feeding cycle. Food intake was measured at 0.5, 1, 2, 3, 6, 16, 24, 48, 72, and 96 h after administration.

The general experimental design for the anorexia studies (Figure 10) was based on protocols developed previously [29]. Briefly, mice were acclimated to the environment and pelleted a high-fat diet for 1 week after arriving, then randomly divided into different groups (*n* = 6/gp) according to body weight. On the day of experiment, mice were fasted from 10:00 h to 18:00 h and provided water ad lib. At 18:00 h, mice were administered 0, 0.01, 0.1, 0.5, and 1 mg/kg BW of T-2, HT-2, DAS, and NEO in 100 µL 1% DMSO by oral gavage, using a sterile 22 G, 1.5 inch disposable feeding tube, or IP injection using a sterile 27 G, 0.5 inch needle. Dose selection was based on preliminary range finding studies. Mice were then immediately provided pre-weighed food pellets. Food intake was monitored for varying lengths of time up to 96 h, depending on the duration of anorexia observed in preliminary experiments. For T-2 and HT-2, food intake was measured at 0.5, 1, 2, 3, 6, 16, and 24 h after oral exposure, or 0.5, 1, 2, 3, 6, 16, 24, 48, 72, and 96 h after IP exposure. For DAS, food intake was measured at 0.5, 1, 2, 3, 6, and 16 h after oral exposure, or 0.5, 1, 2, 3, 6, 16, 24, 48, 72, and 96 h after IP exposure. For NEO, food intake was measured at 0.5, 1, 2, 3, 6, and 16 h after oral exposure, or 0.5, 1, 2, 3, 6, 16, 24, and 48 h after IP exposure.

### 5.4. Statistics

All data were plotted and statistically analyzed using SigmaPlot 11 for Windows (Jandel Scientific; San Rafael, CA, USA). Data were considered to be statistically significant if *p* < 0.05. One-way ANOVA using the Holm-Sidak Method was used to determine the significant differences between treatments and the respective control. Two-way repeated ANOVA (one-factor) using the Holm-Sidak method was used to analyze significant differences in food consumption as compared with the control over time.

## Figures and Tables

**Table 1:** Summary of NOAELs and LOAELs for anorectic effects of T-2 toxin, HT-2 toxin, diacetoxyscirpenol (DAS) and neosolaniol (NEO).

Toxin (mg/kg BW)	Oral		Intraperitoneal	
	NOAEL ^a^	LOAEL ^b^	NOAEL ^a^	LOAEL ^b^
T-2	0.01	0.1	0.01	0.1
HT-2	0.01	0.1	0.01	0.1
DAS	0.1	0.5	< 0.01	0.01
NEO	0.01	0.1	0.01	0.1

^a^ NOAEL = no observed adverse effect level; ^b^ LOAEL = lowest observed adverse effect level.
